# Epstein-Barr Virus-Induced Minimal Change Disease: A Cause of Nephrotic Syndrome in Infectious Mononucleosis

**DOI:** 10.7759/cureus.87315

**Published:** 2025-07-05

**Authors:** Srijeet Ghatak, Prapa Bose

**Affiliations:** 1 Gastroenterology, Ealing Hospital, Croydon, GBR; 2 Internal Medicine, Epsom and St Helier Hospital, Sutton, GBR

**Keywords:** epstein-barr virus, infectious mononucleosis (im), kissing disease, primary nephrotic syndrome, proteinurea

## Abstract

Infectious mononucleosis (IM), caused by the Epstein-Barr virus (EBV), typically presents as a self-limiting illness characterized by fever, pharyngitis, and lymphadenopathy. While subclinical renal involvement is not uncommon, acute kidney injury (AKI) and nephrotic syndrome due to EBV are rare. We present the case of an 18-year-old woman with EBV-induced IM complicated by minimal change disease (MCD), resulting in nephrotic syndrome. The patient responded promptly to steroid therapy, highlighting the importance of considering EBV in the differential diagnosis of minimal change nephropathy.

## Introduction

Epstein-Barr virus (EBV) is a ubiquitous herpesvirus infecting over 95% of the adult population worldwide. Primary infection most commonly manifests as infectious mononucleosis (IM), characterized by fever, pharyngitis, and lymphadenopathy [1]. Renal involvement in IM is typically mild and subclinical, with proteinuria and hematuria reported in approximately 10-15% of cases. However, acute kidney injury (AKI) and nephrotic syndrome are rare complications. Minimal change disease (MCD) is an uncommon but important manifestation of EBV-associated renal disease, with only sporadic cases reported in the literature [2]. We describe a rare case of EBV-associated MCD presenting with nephrotic syndrome, emphasizing the importance of considering EBV infection in the differential diagnosis of nephrotic syndrome, especially when preceded by a viral prodrome.

## Case presentation

An 18-year-old woman presented with a one-week history of fever, increasing fatigue, lightheadedness, and abdominal discomfort. She denied coryza, sore throat, or gastrointestinal symptoms. She reported facial swelling in the mornings and recent polyuria. Her last menstrual period was irregular. She had a small rash on her abdomen one week prior. Physical examination was unremarkable apart from mild facial puffiness. Laboratory investigations revealed a white cell count of 17.4 × 10^9^/L, lymphocytosis (11.9 × 10^9^/L), and mild anemia (Hb 122 g/L) (Table 1). Liver function tests showed elevated alanine aminotransferase (ALT) (282 U/L) and alkaline phosphatase (ALP) (354 U/L), which trended downwards. Urinalysis was notable for 3+ proteinuria, with a protein-creatinine ratio of 20. Viral serology was positive for EBV viral capsid antigen (VCA). The microscopy result of the renal biopsy is mentioned in Table 2.

**Table 1:** Laboratory blood test

Test	Value	Normal Range	Interpretation
White Cell Count (WCC)	17.4 ×10⁹/L	4.0–11.0 ×10⁹/L	Elevated – likely reactive, typical in viral infections
Neutrophils	4.3 ×10⁹/L	2.0–7.5 ×10⁹/L	Normal – argues against bacterial infection
Lymphocytes	11.9 ×10⁹/L	1.0–4.0 ×10⁹/L	Elevated – lymphocytosis, typical in infectious mononucleosis (IM)
Hb (Hemoglobin)	122 g/L	120–160 g/L (female); 130–180 g/L (male)	Mild anemia can be seen with infection/inflammation
Monocytes	0.5 ×10⁹/L	0.2–0.8 ×10⁹/L	Normal
Erythrocyte Sedimentation Rate (ESR)	12 mm/hr	0–20 mm/hr	Mildly elevated – consistent with viral infection
Alanine Aminotransferase (ALT)	282 → 41 U/L	7–56 U/L	Initially elevated, resolving hepatic involvement (viral hepatitis pattern)
Alkaline Phosphatase (ALP)	354 → 74 U/L	44–147 U/L	Initially elevated, resolving cholestatic involvement (seen in viral illness)
Urine Dip (Protein)	3+	Negative to trace	Significant proteinuria – suggests renal involvement
Protein: Creatinine Ratio	20	<15	Elevated – consistent with nephrotic syndrome
Epstein-Barr Virus Viral Capsid Antigen IgM	Positive	Negative	Indicates active or recent EBV infection
Epstein-Barr Virus Viral Capsid Antigen IgG	Positive	Negative or Positive	Indicates past or current EBV infection
EBNA IgG (Epstein-Barr Nuclear Antigen)	Positive	Negative or Positive	Indicates past infection
Blood Film	Reactive lymphocytes, smear cells, giant platelets	Normal lymphocytes, no smear cells	Suggestive of EBV infection, typical blood smear findings
Blood Culture	Negative	Negative	Rules out bacteremia

**Table 2:** Microscopy result of renal biopsy

Test	Result
Light Microscopy	Well-preserved parenchyma and normal appearing glomeruli. Tubular epithelial cells contained protein reabsorption granules.
Electron Microscopy	Extensive effacement (>80%) of the foot processes of the visceral epithelial cells, consistent with minimal change disease.

This patient was started on the steroid methylprednisone at 16 mg and was titrated by 4 mg every week. For three weeks, this patient was given methylprednisolone, and when on only 4 mg, was brought back to the ambulatory care unit (ACU), and a repeat urine protein was done, which showed levels had returned to normal.

Complete resolution was achieved and continued for another week at 4 mg, after which it was stopped. She was brought into the ACU four weeks after her steroid therapy, and it was seen that her urine protein-to-creatinine ratio was still normal, and the urine dip showed no protein, hence complete resolution even after stopping steroids.

## Discussion

EBV, a ubiquitous herpesvirus, is best known for causing IM. While its typical clinical manifestations include fever, pharyngitis, and lymphadenopathy, EBV can also cause a wide spectrum of systemic complications, including renal involvement, although this is relatively rare [1-3].

Among the renal complications, MCD is an especially uncommon but clinically important manifestation. The majority of MCD cases are idiopathic; however, secondary forms have been associated with infections, malignancies, and drugs [2,3]. When MCD occurs in the context of a viral illness like EBV, it poses a diagnostic and therapeutic challenge, especially in the absence of classic symptoms of IM, as in our case.

**Figure 1:**
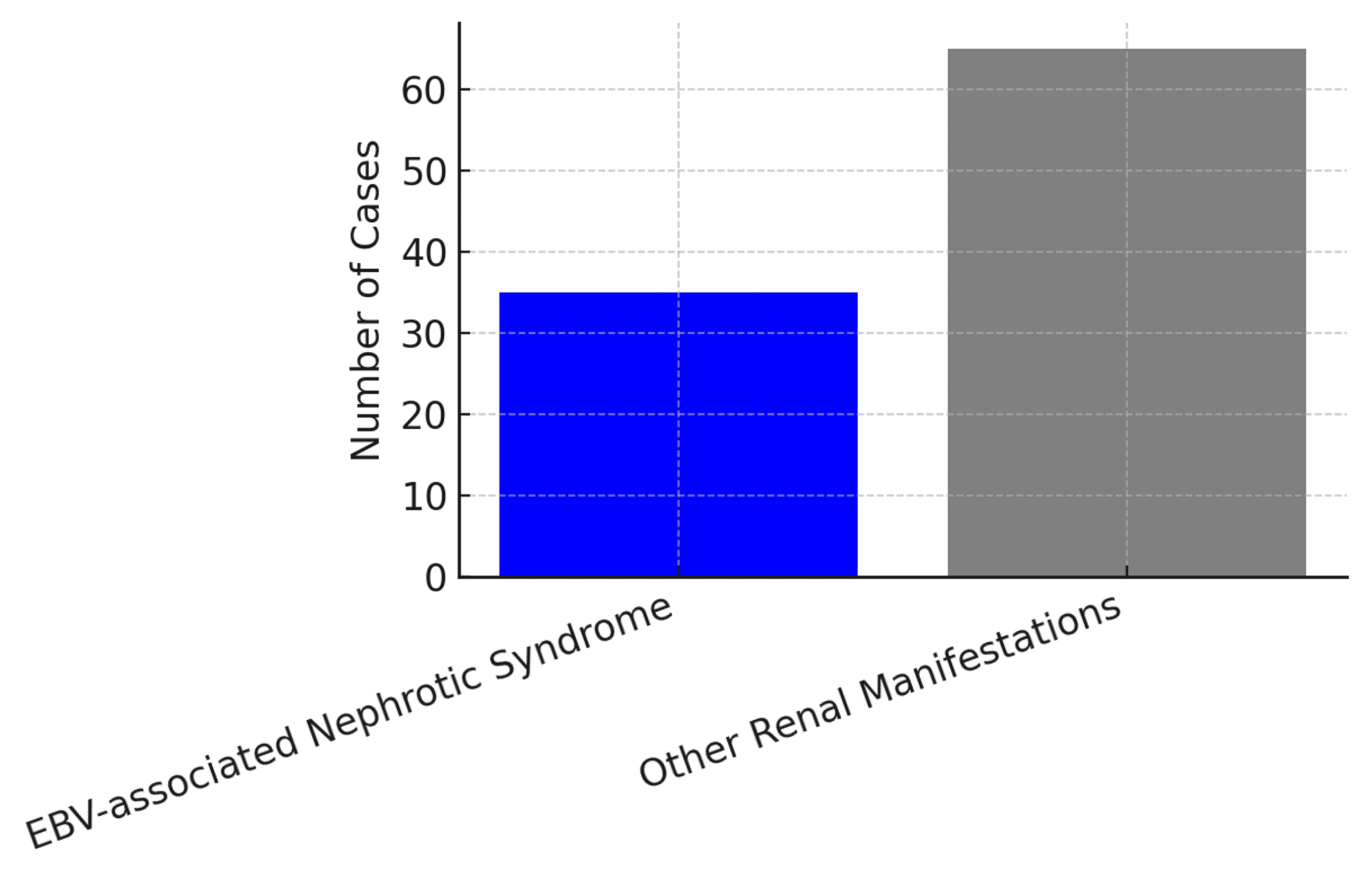
EBV-associated other renal disease as compared to nephrotic syndrome in 100 cases EBV: Epstein-Barr Virus

Figure 1 shows the rarity of nephrotic syndrome in a total of 100 patients with renal disease secondary to EBV; 65 had other renal manifestations, and 35 had nephrotic syndrome.

The proposed mechanisms by which EBV may contribute to renal pathology include both immune-mediated and direct viral mechanisms. One theory suggests that the virus triggers a T-cell-mediated response that causes the release of cytokines, ultimately affecting glomerular permeability and leading to podocyte injury [3-5]. Another plausible mechanism involves direct viral cytopathic effects. Studies have demonstrated EBV DNA within renal biopsy specimens and have shown that renal tubular epithelial cells express the CD21 receptor, which EBV uses for cellular entry, supporting the idea of direct infection of renal tissue [4,6,7].

Histologically, MCD is defined by the effacement of podocyte foot processes seen on electron microscopy, while light microscopy often reveals normal-appearing glomeruli [2,6,8]. This underscores the critical importance of renal biopsy in atypical presentations of nephrotic syndrome, as it was essential in our case to reach a definitive diagnosis and guide treatment.

Our patient demonstrated rapid clinical improvement following corticosteroid therapy, a response that is well-documented in MCD regardless of its etiology [2]. The patient was started on dexamethasone 10 mg on day one, followed by a tapering dose of methylprednisolone beginning at 16 mg per day and tapered by 4 mg daily to zero for persistent proteinuria. While the use of immunosuppression in active EBV infection raises concerns about potential viral reactivation, multiple case reports and small case series suggest that the timely initiation of corticosteroids in EBV-associated MCD can lead to favorable outcomes without precipitating severe viral complications [1,6,9].

Furthermore, renal involvement may be under-recognized in the setting of EBV infection, particularly in adults who present atypically. This highlights the need for a high index of suspicion in patients presenting with nephrotic-range proteinuria in the setting of recent or ongoing viral illness, even in the absence of IM’s classical clinical features [6,9].

Finally, EBV has also been implicated in other glomerular diseases such as collapsing glomerulopathy and membranous nephropathy, emphasizing its capacity to affect renal tissues in diverse ways [4,9,10]. Continued reporting of EBV-associated renal pathology is necessary to better understand its spectrum and pathogenesis and to inform appropriate diagnostic and treatment strategies.

## Conclusions

MCD is a rare but significant renal complication associated with EBV-induced IM. While EBV is best known for causing fever, lymphadenopathy, and hepatosplenomegaly, its potential to cause immune-mediated glomerular injury is often underrecognized. MCD typically presents with features of nephrotic syndrome, including heavy proteinuria, hypoalbuminemia, and edema. Clinicians should maintain a high index of suspicion for EBV as an underlying etiology in patients who present with new-onset nephrotic syndrome, particularly when there is a recent history of viral illness or prodromal symptoms such as fatigue, sore throat, or malaise. Diagnosis may be supported by EBV serology and clinical context, with renal biopsy considered in uncertain cases. Importantly, MCD associated with EBV generally responds well to corticosteroid therapy, and early recognition can significantly improve renal outcomes. Awareness of this association allows for timely treatment and avoids unnecessary delay in initiating appropriate management.
